# Orthotopic Heart Transplantation and Mechanical Circulatory Support in Cancer Survivors: Challenges and Outcomes

**DOI:** 10.1155/2015/232607

**Published:** 2015-08-03

**Authors:** Nina Ghosh, John Hilton

**Affiliations:** ^1^Ottawa Cardiovascular Centre, Cardio-Oncology Survivorship Clinic, 1355 Bank Street, Suite 502, Ottawa, ON, Canada K1H 8K7; ^2^Division of Oncology, Department of Medicine, University of Ottawa Cancer Centre, 501 Smyth Road, Ottawa, ON, Canada K1H 8L6

## Abstract

Chemotherapy-induced cardiomyopathy (CCMP) is a significant cause of morbidity and mortality. Compared to cardiomyopathy due to other causes, anthracycline-induced cardiomyopathy is associated with a worse survival. As cancer survival improves, patients with CCMP can be expected to comprise a significant proportion of patients who may require advanced therapies such as inotropic support, cardiac transplantation, or left ventricular assist device (LVAD). Distinct outcomes related to advanced therapies for end-stage heart failure in this patient population may arise due to unique demographic characteristics and comorbidities. We review recent literature regarding the characteristics of patients who have survived cancer undergoing orthotopic heart transplantation and mechanical circulatory support for end-stage heart failure. The challenges and outcomes of advanced therapies for heart failure related specifically to anthracycline-induced cardiomyopathy are emphasized.

## 1. Introduction

Chemotherapy-induced cardiomyopathy (CCMP) is a significant cause of morbidity and mortality [1]. Compared to cardiomyopathy due to other causes including idiopathic cardiomyopathy and ischemic cardiomyopathy, anthracycline-induced cardiomyopathy is associated with a worse survival [2]. Up to 2–4% of patients with anthracycline-induced cardiomyopathy progress to end-stage heart failure, a proportion of who may require advanced therapies such as inotropic support, orthotopic heart transplantation (OHT), or left ventricular assist device (LVAD) [1]. As such, the feasibility of advanced therapies is an important consideration in this patient population [1].

Distinct outcomes related to advanced therapies for end-stage heart failure in this patient population may arise due to unique demographic characteristics and comorbidities. Furthermore, there are pathophysiological differences between anthracycline-induced cardiomyopathy and other causes of cardiomyopathy that may impact feasibility of isolated left ventricular support. Indeed, the mechanisms of cardiac injury after anthracycline-induced chemotherapy are significantly different from those involved in ischemic or idiopathic/dilated cardiomyopathy [3]. Anthracycline-induced cardiomyopathy is thought to be mediated by a variety of mechanisms including the production of free radicals, mitochondrial damage, and mitochondria-dependent apoptosis. Doxorubicin has also been shown to intercalate DNA by forming complexes with topoisomerase II beta. The resulting histological findings include cytoplasmic vacuolization, myofibril loss and disarray, cellular necrosis and fibrosis [4, 5]. It must be noted, however, that the diagnosis of anthracycline-induced cardiomyopathy in most studies has been presumed based on clinical history and exclusion of other causes. Right ventricular endomyocardial biopsy with the cardinal findings of anthracycline-induced cardiotoxicity is the gold standard for confirming anthracycline-induced cardiotoxicity as the cause of cardiomyopathy.

The case illustration introduces several important challenges that clinicians who are caring for patients with end-stage heart failure due to cancer treatment face. These include the implications of the oncologic prognosis on heart failure treatment options and the impact of prior cancer treatment on perioperative and longer-term outcomes after LVAD implantation or OHT.

Important concepts related to the advanced heart failure in cancer patients have been recently reviewed [6, 7]. Shah and Nohria highlight the pathophysiology, clinical presentation, and management of heart failure due anthracyclines, targeted therapies, and restrictive cardiomyopathy secondary to thoracic radiation [6]. Importantly, the review outlines preventative strategies and pharmacological management of left ventricular dysfunction due to cancer treatment. Oliveira et al. recently critically appraised the data available for advanced therapies including implantable cardioverter defibrillator, cardiac resynchronization therapy, OHT, and mechanical circulatory support (MCS) for chemotherapy-induced cardiomyopathy [7].

This paper will specifically focus on the current knowledge of the epidemiology and outcomes related to OHT and MCS in patients with a history of cancer with a focus on patients with presumed anthracycline-induced cardiomyopathy. Herein, CCMP and anthracycline-induced cardiomyopathy will be used interchangeably.

## 2. Case Illustration

A fifty-seven-year-old female is admitted to hospital with symptoms of congestive heart failure including dyspnea, orthopnea, and paroxysmal dyspnea. Echocardiography shows severely globally reduced left ventricular ejection fraction (LVEF) of 10–15% with mild dilation, moderately reduced right ventricular function, moderate mitral regurgitation, and severe tricuspid regurgitation. Coronary angiography shows no evidence of coronary atherosclerosis. She undergoes further diuresis and is started on low dose ace-inhibitors and digoxin and is subsequently discharged home in stable condition.

Eleven months prior, she underwent treatment with 4 cycles of adjuvant Adriamycin/Cytoxan chemotherapy after mastectomy and radiation to the right chest for T2N3M0, estrogen receptor negative/progesterone receptor negative/HER2 negative “triple negative”, breast cancer. She received a total dose of 240 mg/m^2^ of Adriamycin for breast cancer. Prior to initiation of chemotherapy for breast cancer, echocardiography showed normal LVEF (>55%).

At the age of 22, she was diagnosed with non-hodgkin's lymphoma (NHL) involving the chest, abdomen, and pelvis for which she was treated with Adriamycin-based chemotherapy and partial small bowel resection. The dose of Adriamycin given during this treatment was unknown.

Three months following her initial heart failure admission, she presents again with decompensated heart failure. Right heart catheterization reveals a cardiac index of 1.4, right atrial pressure of 16 mmHg, pulmonary artery pressure of 46/22 mmHg (30), pulmonary capillary wedge pressure of 20 mmHg, and systemic vascular resistance of 2000 dyn∗s/cm^5^. Milrinone is initiated with symptomatic improvement, an increase in cardiac index to 2.1 and a decrease in systemic vascular resistance to 1065 dyn∗s/cm^5^. During this hospitalization, the Advanced Heart Disease service is consulted for consideration of advanced therapies for heart failure. To guide decision-making, the patient's cancer prognosis is discussed with the patient's Oncologist who quotes a risk of recurrence of breast cancer of >50% in the next 3 to 5 years. After careful discussion with the patient and members of her multidisciplinary clinical team, the decision is made to discharge the patient on home intravenous milrinone therapy.

The patient remains stable on home intravenous milrinone therapy for one month. However, she represents to an outside hospital with a 20 lb weight gain, abdominal bloating, orthopnea, and paroxysmal dyspnea. Intravenous dobutamine is added to her regimen. The patient is transferred to a tertiary care hospital for consideration of destination therapy LVAD. After transfer, the patient undergoes further diuresis, and with dobutamine at 2.5 mcg/kg/min and milrinone at 0.5 mcg/kg/min being administered, she undergoes right heart catheterization (RHC). RHC shows a central venous pressure of 22 mmHg, pulmonary artery pressure of 38/22 (27), pulmonary capillary wedge pressure of 25 mmHg, cardiac index of 2.5, and arterial blood pressure of 72/48 mmHg. Hemodynamics is further optimized by increasing dobutamine to 4 mcg/kg/min further diuresis, and insertion of an intra-aortic balloon pump. CVP improves to 14 mmHg and PCWP to 14 mmHg. On the same day, the patient undergoes implantation of Thoratec's HeartMate II left ventricular assist device. After LVAD implantation, she shows signs of persistent right ventricular failure including central venous pressure in the low to mid 20's mmHg despite adequate left ventricular unloading. She is taken back to the operating room for the TandemHeart right ventricular assist device. Her postoperative course is further complicated by acute kidney injury and acute respiratory distress syndrome. On postoperative day 13, she is found to be unresponsive. Computed tomography shows massive intracranial hemorrhage. The family decides to transition her care philosophy to comfort measures only. The patient dies peacefully, surrounded by her family, on postoperative day 14.

## 3. Orthotopic Heart Transplantation (OHT)

OHT in survivors of cancer carries the potential risk of relapse of the primary malignancy, leading to the concern for poorer prognosis after OHT. The concern for post-OHT malignancy is greater for cardiac transplantation compared to other solid organ transplants such as renal transplantation because of a relatively increased level of immunosuppression required due to less extensive HLA matching [8].

Current guidelines state that a history of active cancer in the form of a solid tumor or hematological malignancy within five years is a contraindication to heart transplantation [9]. For those who are beyond this window, recent evidence involving larger patient cohorts [10–13] suggests that outcomes for both pediatric and adult survivors of cancer who develop end-stage heart failure are comparable to recipients without a history of cancer.

In a recent study of 7169 pediatric heart transplant recipients who were identified using the UNOS (United Network for Organic Sharing) database, several important observations were made. Of the overall cohort, 1.5% had a history of childhood cancer (*N* = 107). Of these 107 patients, 25% had a history of leukemia. The incidence of post-OHT malignancy was significantly higher in recipients with a pre-OHT history of cancer than those without (13% versus 5.4%, resp., *p* < 0.001). Despite the increased risk of posttransplant malignancy, overall survival at one year and at five years was similar in the two groups (90.6% and 80.3%, resp., in the cancer group, and 84.4% and 73.8%, resp., in the noncancer group, *p* < 0.001). Survival was also the same when only patients, with cardiomyopathy (rather than congenital heart disease) as the reason for transplantation, were compared [14].

Similar outcome patterns have been observed in adult patients with a history of cancer undergoing OHT. Oliveira et al. looked specifically at characteristics and outcomes of adult patients undergoing OHT for presumed CCMP [1]. The patient cohort was derived from the International Society of Heart and Lung Transplantation (ISHLT) Registry, from which 232 heart transplant patients aged 18 or older who carried a diagnosis of CCMP were identified. This group was compared to a control group of 8890 patients with NICM who underwent OHT during the same time period. The most common malignancies were hematologic (33%), followed by breast cancer (31%) and sarcomas (7.5%), malignancies for which anthracyclines are frequently the mainstay of therapy.

Important baseline differences were observed between the groups. First, a significantly greater proportion of the CCMP group was females compared to the NICMP group that was females (65% females in the CCMP group versus 25% females in the NICM group, *p* < 0.001). This is despite the fact that hematologic malignancies and sarcomas comprised a greater proportion of cancers (44%) compared to breast cancer (31%), raising the possibility that women may be more vulnerable to anthracycline-induced cardiomyopathy than men [1]. Interestingly, the CCMP group had a greater need for right ventricular assist device prior to transplantation compared to the NICM group, a finding that may have important implications for both the underlying pathophysiology of CCMP and the feasibility of isolated LVAD in this patient population. This will be further discussed in the section reviewing MCS below.

As observed in the pediatric study, the post-OHT malignancy rate was significantly greater in the CCMP group compared to the control group (5% in the CCMP group versus 2% in the NICMP group, *p* = 0.006) [1]. The majority of the cancers in both groups were nonfatal skin cancers with only one patient having recurrence of his pre-OHT malignancy (breast cancer). Despite these differences in post-OHT malignancy rates, post-OHT short-term and long-term survival rates were similar between the two groups. At 1 and 5 years, survival in the CCMP group was 86% and 71%, respectively. In the NICMP group, survival at 1 and 5 years was 87% and 74%, respectively [1]. Furthermore, there was no statistically significant difference in malignancy as being the cause of death in either group at 1 and 5 years.

In summary, the evidence outlined above suggests that both children and adults with CCMP prior to OHT have comparable and favorable survival compared to those with other causes of end-stage heart disease. This is despite higher rates of malignancy post-OHT. Indeed, the malignancies were rarely due to recurrence of the original malignancy. The study by Oliveira et al. also suggests that patients who undergo OHT for CCMP have a lower burden of comorbidities including renal dysfunction, hypertension, and diabetes [1].

Some outstanding questions remain. What is the underlying cause of greater rates of malignancy in patients undergoing OHT after transplantation? Does it reflect overzealous post-OHT immunosuppression or does it suggest residual intrinsic immunodeficiency after treatments with potential toxicity to the bone marrow and other immune-modulating agents? Does the lower rate of comorbidities reflect stricter selection for this patient population on the part of the treating clinicians or does it simply reflect the general profile of patients considered for transplantation?

Overall, OHT outcomes in patients with a history of cancer and probable CCMP are favorable with survival similar to other OHT recipients. Therefore, anthracycline treatment or a history of cancer should not preclude consideration of OHT particularly if the cancer has been in remission for five years or more as endorsed by current guidelines [15]. Oliveira et al. suggest that the 5-year post-remission OHT eligibility criterion should be replaced by eligibility on a case-by-case basis in consultation with the oncology team [7]. Prospective studies are required to support the safety and feasibility of this approach.

## 4. Mechanical Circulatory Support (MCS)

Unlike other causes of cardiomyopathy, OHT may be contraindicated due to coexistence of malignancy or timing of malignancy remission. As such, when conventional therapies for heart failure have been exhausted, MCS such as LVAD may remain the only option outside of supportive care. This section will review the current literature regarding the epidemiology, outcomes, and specific challenges associated with LVAD as both destination therapy (DT) and bridge to transplantation (BTT) in patients with CCMP.

*Characteristics of Patients Undergoing LVAD Implantation*. Until recently, there was a paucity of data regarding MCS in patients with end-stage heart failure due to anthracycline-induced cardiomyopathy. Several case reports describe LVAD implantation serving as a bridge to recovery of cardiac function in presumed anthracycline-induced cardiomyopathy, refuting the widely held concept that anthracycline-induced cardiomyopathy is irreversible (the so-called type I cardiotoxicity) [16–18]. LVAD as bridge to recovery is expected in forms of cardiomyopathy with a reversible trajectory such as fulminant myocarditis and other forms of nonischemic dilated cardiomyopathy. Hypotheses for the unexpected recovery after LVAD implantation in these reports include concurrent implementation of measures that could lead to reverse remodeling such as aggressive use of beta blockers and angiotensin-converting-enzyme inhibitors, cardiac resynchronization therapy, LV unloading prior to the necrotic and fibrotic stages of anthracycline-induced cardiomyopathy, and misdiagnosis of the etiology of cardiomyopathy. Although these reports are of interest and suggest a partially reversible potential of anthracycline-induced cardiomyopathy, they do not give insight into the overall success rate and outcomes of MCS.

Recently, Oliveira et al. reported on the characteristics and outcomes of a cohort of patients with CCMP undergoing MCS [19]. The authors queried The Interagency Registry for Mechanically Assisted Circulatory Support (INTERMACS) to retrospectively identify patients with presumed CCMP cardiomyopathy who underwent MCS. Participation in this registry is mandatory in the United States for MCS implantation centers to gain approval from the Centers for Medicare and Medicaid Services. Between June 2006 and March 2011, the authors were able to identify 75 patients (2%) with CCMP out of 3812 with other causes of cardiomyopathy.

The baseline characteristics of CCMP patients undergoing MCS therapy paralleled those of CCMP patients undergoing OHT. The majority of CCMP patients (52%) had a history of breast cancer and 33% had a history of lymphoma or another hematological cancer. CCMP patients undergoing implantation of MCS devices were much more likely to be females (72% in the CCMP group versus 24% and 13%, resp., in the NICM and ICM groups) and had less comorbidity such as diabetes and tobacco use. Interestingly, the use of ICDs was significantly lower in the CCMP group than in the ICMP and NICM groups [19].

The longitudinal therapeutic goals of MCS support can be classified as follows: a bridge to cardiac transplantation, a bridge to cardiac recovery, lifelong therapy (also referred to as “destination” therapy), and a bridge to candidacy for cardiac transplantation. In the cohort studied by Oliveira et al., a greater proportion of patients with CCMP underwent MCS implantation as destination therapy than did patients with NICMP and ICMP [19]. Importantly, CCMP patients undergoing MCS implantation as destination therapy had significantly reduced survival compared to those receiving MCS as BTT (32% death rate versus 22% death rate in DT versus BTT patients, resp.) [19]. Prior studies have shown that a history of solid organ malignancy is associated with up to 89% greater likelihood of early death after DT LVAD implantation compared to those without a history of solid organ malignancy [20]. Why a history of malignancy in recipients of destination therapy MCS is associated with a greater risk of death requires further exploration. Specifically, elucidating whether poorer survival is related to competing cancer mortality, a higher burden of comorbidities, frailty, nutritional status, or greater risks of post-implantation complications such as bleeding, infection, and thrombosis will be important to guide patient selection for this costly therapy.

## 5. Need for Right Ventricular Assist Device (RVAD)

Oliveira et al. showed that a significantly greater proportion of CCMP patients required RVAD either concomitantly or following LVAD implantation. Specifically, 19% of CCMP versus 9.3% of non-CCMP patients (*p* < 0.001) required RVAD perioperatively [19]. The need for greater right ventricular failure in this patient population may be due to several causes.

First, it may reflect the pathophysiological process underlying anthracycline-induced cardiomyopathies, including proportionate deterioration of RV and LV functions in anthracycline-induced cardiomyopathy compared to the LV predominance observed in other causes of cardiomyopathy. The biventricular nature of anthracycline-mediated cardiomyopathy has been demonstrated in cardiac imaging studies [21–23]. Poorer right ventricular function around the time of MCS implant may also reflect predilection for clinicians to delay advanced therapies in patients with a history of recently treated cancer. Finally, poorer RV function may reflect higher incidences of pulmonary hypertension related to prior pulmonary emboli in patients with a history of cancer. This hypothesis was not supported by the Oliveira et al. study, which showed similar pulmonary vascular resistance in the CCMP group and the NICMP/ICMP groups prior to MCS implantation. Nevertheless, the important observation of greater need for RVAD in CCMP patients emphasizes the need for vigilance regarding the status of the right ventricle on the part of clinicians caring for patients with anthracycline-induced cardiomyopathy. This is particularly important because biventricular mechanical support is currently not feasible or approved as destination therapy and morbidity and mortality increase significantly with right ventricular failure after LVAD implantation.

Despite higher rates of post-MCS bleeding rates and a greater need for RVAD in the CCMP group, Oliveira et al. showed similar overall survival between all groups.

## 6. Surgical Considerations to MCS in Patients with Chemotherapy-Induced Cardiomyopathy

The feasibility of MCS may be affected by several technical considerations. Median sternotomy is required for the implantation of most contemporary continuous flow LVADs including Heart Mate II (Thoratec Corporation; Pleasanton, CA) and HeartWare (HeartWare International, Inc., Framingham, MA). Prior radiation therapy is an important consideration for sternotomy in CCMP patients being considered for MCS. A history of thoracic radiation therapy itself is a risk factor for developing cardiomyopathy after anthracycline therapy [24, 25]. High-dose thoracic field radiation therapy for breast cancer and lymphoma can lead to severe postradiation sternal damage. Under these circumstances, median sternotomy may be associated with a prohibitive risk of surgical complications including postoperative deep sternal wound infections [26]. Because there is a broad spectrum of mediastinal radiation-induced injury, ranging from minor fibrosis to heavy scarring and fusion of mediastinal structures including pericardial, myocardial, vascular and valvular damage, the extent of this injury should be taken into consideration in patients with a history of prior radiation therapy [27]. Although experience with minimally invasive, off-pump approaches to LVAD implantation is increasing, particularly with HeartWare devices [26], such approaches are not currently approved and are limited by the frequent need to address coexisting cardiac lesions including tricuspid and aortic insufficiency and patent foramen ovale/atrial septal defect at the time of LVAD implantation.

## 7. Patient Size and Body Habitus

A potentially relevant observation in studies of LVAD and OHT in CCMP patients is the significantly lower body surface area (BSA) in patients with CCMP compared to those with other forms of cardiomyopathy. BSA was 12% lower in patients with CCMP undergoing LVAD implantation in the study by Oliveira et al. (BSA was 1.84 m^2^ in the CCMP group versus 2.09 m^2^ in the group with other forms of cardiomyopathy, *p* < 0.0001) [19]. Several factors may have contributed to this observation including the greater proportion of women and the effects of previous cancer treatment on growth in the case of pediatric acute lymphocytic leukemia. Indeed, growth deficit is a known complication of both anthracycline therapy and cranial irradiation in pediatric acute lymphocytic leukemia survivors [28]. Implantation of LVADs was previously restricted to patients with a body surface area >1.5 m^2^. Fortunately, the continuous flow LVADs have demonstrated a good safety profile in patients as with BSA that is as low as 1.3 m^2^ allowing for the use of nonpulsatile VADS in a significantly higher proportion of women, smaller adults, and adolescents [29]. Furthermore, the HeartWare device, which is implanted within the pericardium, has essentially eliminated small BSA restrictions to LVAD implantation [29]. Nevertheless, lower BSA has been shown to be associated with poorer outcomes and higher stroke risk after continuous flow LVAD implantation [30]. Finally, the observation of greater rates of right ventricular failure in this patient population makes Total Artificial Heart (TAH, SynCardia Systems Inc., Tucson, AZ) an important consideration. However, SynCardia Systems, Inc., recommends a minimum patient body surface area (BSA) of 1.7 m^2^ [31]. Future studies are needed to determine if there is an important proportion of patients with a history of anthracycline-induced chemotherapy that are excluded from MCS due to small body surface area or, on the other hand, whether lower BSA reflects lower rates of obesity.

## 8. Conclusions and Future Directions

This case demonstrates the potential and unique challenges associated with the management of advanced heart failure in patients with a history of cancer and probable anthracycline-induced chemotherapy. The oncologic prognosis influenced the decision to defer LVAD and pursue home IV inotropes. However, when faced with another acute decompensation, LVAD was implanted but at a point when right ventricular dysfunction had progressed significantly. In retrospect, several clinical and hemodynamic parameters have been shown to predict right heart failure (such as elevated central venous pressure-to-pulmonary capillary wedge pressure ratio and right ventricular dysfunction on echocardiography) were present [32–35]. It remains unclear whether anthracycline-based chemotherapy in and of itself is a risk factor for right ventricular failure. It is possible that preemptive RVAD or TAH implantation could have mitigated her clinical demise. TAH was not an option as its use is approved only as a bridge to transplantation. TAH was also not an option due to her small body habitus. Finally, options related to end-of-life care such as nonhospice palliative care or hospice care could have been presented at an earlier point in the patient's clinical course. Therefore, this case exemplifies many of the complexities associated with advanced heart failure in patients with a history of cancer, particularly those who are not transplant candidates.

As a whole, patients with CCMP appear to have similar survival after OHT/LVAD therapy, respectively, compared to patients with other etiologies of cardiomyopathy. This is despite higher rates of malignancy after OHT and a greater need for RVAD after LVAD implantation. A history of cancer and end-stage heart failure should not preclude thoughtful and timely consideration of advanced therapies for end-stage heart failure. Rather, a comprehensive multidisciplinary approach that integrates the wishes and values of the patient with anticipation of potential complications should facilitate optimal care. Based on the current body of evidence, the various factors that may influence decision-making and outcomes after OHT and LVAD for anthracycline-induced end-stage heart failure are summarized in Table 1.

It is important to note that the overall evidence presented in this review is limited both by the very small number of studies and by retrospective nature of these studies. Furthermore, there is a paucity of data on outcomes of MCS in patients who are not eligible for transplant. In anticipation of a growing number of patients surviving long enough following cancer treatment to experience the cardiotoxic side effects of their cancer treatment, advanced therapies for end-stage heart failure in this patient population should be an intense focus of future research.

## Figures and Tables

**Table 1:** Factors that may influence outcome after advanced heart failure therapies in adult patients with anthracycline-induced cardiomyopathy [1].

Patient characteristics that may favor advanced heart failure therapies in anthracycline-induced end-stage heart failure	Patient characteristics that may oppose advanced heart failure therapies in anthracycline-induced end-stage heart failure
Lower body surface area	Higher rates of malignancy^*^
Lower likelihood of diabetes	Higher rates of infection
Lower likelihood of hypertension	Impact of prior thoracic radiation therapy on sternotomy complications
Lower likelihood of smoking	Greater need for right ventricular assist device
	Higher rates of perioperative bleeding
Younger age	Higher rates of destination-therapy (as opposed to bridge to transplantation)^#^

^*^Applies to orthotopic heart transplantation but not left ventricular assist device.

^#^Applies to left ventricular assist device but not to orthotopic heart transplantation.
